# COVID-19 Pandemic: Insights into Interactions between SARS-CoV-2 Infection and MAFLD

**DOI:** 10.7150/ijbs.72461

**Published:** 2022-07-11

**Authors:** Hanfei Chen, Qiang Chen

**Affiliations:** 1 Cancer Center, Faculty of Health Sciences, University of Macau, Taipa, Macau SAR, China.; 2 Centre for Precision Medicine Research and Training, Faculty of Health Sciences, University of Macau, Taipa, Macau SAR, China.; 3 MOE Frontier Science Centre for Precision Oncology, University of Macau, Taipa, Macau SAR, China.

**Keywords:** COVID-19, SARS-CoV-2, MAFLD, NAFLD, Liver injury

## Abstract

COVID-19, caused by severe acute respiratory syndrome coronavirus 2 (SARS-CoV-2), has become an ongoing global health pandemic. Since 2019, the pandemic continues to cast a long shadow on all aspects of our lives, bringing huge health and economic burdens to all societies. With our in-depth understanding of COVID-19, from the initial respiratory tract to the later gastrointestinal tract and cardiovascular systems, the multiorgan involvement of this infectious disease has been discovered. Metabolic dysfunction-associated fatty liver disease (MAFLD), formerly named nonalcoholic fatty liver disease (NAFLD), is a major health issue closely related to metabolic dysfunctions, affecting a quarter of the world's adult population. The association of COVID-19 with MAFLD has received increasing attention, as MAFLD is a potential risk factor for SARS-CoV-2 infection and severe COVID-19 symptoms. In this review, we provide an update on the interactions between COVID-19 and MAFLD and its underlying mechanisms.

## Introduction

The coronavirus outbreak, caused by severe acute respiratory syndrome coronavirus 2 (SARS-CoV-2), led to the COVID-19 pandemic and has placed an unprecedented burden on our world, including health systems, social life, economics, and infrastructure 1, 2. SARS-CoV-2, belonging to the Coronaviridae family with SARS-CoV and Middle East respiratory syndrome-related coronavirus (MERS-CoV), consists of a positive single-stranded RNA genome of approximately 30 kb 3-5. SARS-CoV-2 shares 82% genome sequence similarity with SARS-CoV and 50% genome sequence homology with MERS-CoV 6. As of the time of writing, there have been 528.8 million confirmed cases worldwide according to the WHO Coronavirus (COVID-19) Dashboard, and this number continues to increase. Statistically, approximately 119 people per 10,000 people die of COVID-19, which is possibly 10 times or more than the mortality rate of influenza. COVID-19 can cause multiple organ involvement in the body, and its symptoms vary from person to person, normally including fever, headache, and dyspnea 7, 8. Symptoms of gastrointestinal involvement, such as nausea, vomiting, and diarrhea, and dysfunction of taste and smell have also been reported 9-11. Approximately 5% of patients experience severe symptoms such as respiratory failure, shock and multiple organ dysfunction 12. More than 50% of COVID-19 patients have reported underlying medical conditions, and approximately one-third have multiple comorbidities 13, which is associated with the high mortality of COVID-19.

Metabolic dysfunction-associated fatty liver disease (MAFLD), formerly known as nonalcoholic fatty liver disease (NAFLD), has been recently recognized by global experts 14, 15. MAFLD is the most common cause of chronic liver disease and it affects approximately 25% of the adult population worldwide 16, 17. The prevalence of MAFLD is expected to increase by 30% by 2030 as the prevalence of obesity, diabetes and metabolic syndrome continues to grow, as well as changes in people's lifestyle and eating habits continue 18. MAFLD is characterized by hepatic steatosis with metabolic dysfunction. Without timely intervention, MAFLD may progress from nonalcoholic steatohepatitis (NASH) to liver cirrhosis and eventually hepatocellular carcinoma (HCC) 19, 20. Liver injury from both SARS-CoV and MERS-CoV has been reported in past SARS and MERS pandemics 21-24. Unlike the heart, lung, and gastrointestinal damage caused by SARS-CoV-2, the clinical significance of liver involvement has been controversial since the beginning of the COVID-19 pandemic 6, 25-30. Increasing evidence has indicated that hepatic microvesicular steatosis and elevations in transaminase and bilirubin occur in COVID-19 patients. It is plausible to believe there is a link between SARS-CoV-2 infection and MAFLD, which has been proposed and discussed in previous studies 31-33. In this review, we provide an update on the epidemiology of COVID-19 patients with liver dysfunction, its potential mechanisms, and the management approaches for MAFLD.

## COVID-19 and liver dysfunction

Although the exact effect of COVID-19 on the liver is currently unclear, it can be noted that liver biochemistry abnormalities appear to be common in COVID-19 patients, affecting approximately 17-58% of individuals with COVID-19 34-48 (Table 1). Liver biochemistry abnormalities manifest primarily as mild and moderate elevations of alanine aminotransferase (ALT) and/or aspartate aminotransferase (AST) in the early stages of the disease 25, 48-50. A small number of patients may have increased serum bilirubin levels and decreased serum albumin levels, and rarely, increased levels of markers of bile duct damage, such as alkaline phosphatase (ALP), gamma-glutamyl transferase (GGT), and total bilirubin (TBIL) 51-53. This suggests that liver injury caused by SARS-CoV-2 infection is mainly hepatocyte damage rather than cholestasis.

In addition to the liver function indices, histopathological examination of COVID-19 patients also suggests a link between COVID-19 and liver dysfunction. The first autopsy report of a patient who died of severe SARS-CoV-2 infection showed moderate microvesicular steatosis and mild lobular and portal venous activity in liver tissue 54. Subsequently, other COVID-19 autopsies showed similar results, with Kupffer cell activation observed in addition to hepatic steatosis and vascular changes 35, 55-57. In addition, liver biopsies from a cohort of 48 deceased COVID-19 patients revealed extensive luminal thrombosis at the portal and sinusoidal levels, as well as portal fibrosis with marked pericyte activation 58.

COVID-19 patients also exhibited more severe liver dysfunction 6, 48. In an initial clinical investigation based on 99 cases, one COVID-19 patient developed severe hepatitis with ALT and AST levels of 7,590 U/L and 1,445 U/L, respectively 49. Liver injury is much worse in severe COVID-19 patients than in patients with mild symptoms 25. This suggests that comorbidity with liver disease in patients with SARS-CoV-2 infection could exacerbate COVID-19 disease and even lead to death. Singh et al. investigated the interaction of preexisting liver disease and COVID-19. Based on a large, diverse cohort of 2,780 COVID-19 patients in the United States, this study indicated that liver abnormalities were found in the vast majority of patients regardless of preexisting liver disease, but patients with liver disease were at higher risk for hospitalization and mortality 59. Moreover, although abnormal liver function in most COVID-19 patients recovers after the course of the disease, permanent liver damage has been reported in severe COVID-19 cases 6, 49, 60. As MAFLD is the most common cause of chronic liver disease, more attention needs to be paid to patients with MAFLD, not only to prevent these patients from being infected with SARS-CoV-2 but also to follow up the outcomes of infected patients.

## SARS-CoV-2 tropism of the liver

The extent to which organ-specific pathology correlates with direct viral replication or consequent immune and cardiovascular complications is clinically relevant. SARS-CoV-2 preferentially infects respiratory cells, but it can be detected in multiple other organs, including the liver 61-63, indicating a multiple-organ tropism of SARS-CoV-2. Notably, there are differences in the viral load and distribution across patients 7, which may require data from larger cohorts to illustrate the distribution of SARS-CoV-2 in different organs.

The establishment of viral tropism depends on the susceptibility and permissiveness of a particular host cell. Studies have found that the entry of SARS-CoV-2 into cells is dependent on the expression of angiotensin-converting enzyme 2 (ACE2) 64, 65. The receptor-binding domain (RBD) of the SARS-CoV-2 spike (S) protein has a high affinity for ACE2, and to achieve its function, SARS-CoV-2 binds to ACE2 to target cells 66. Transmembrane serine protease 2 (TMPRSS2) in host cells further facilitates viral uptake by cleaving ACE2 and activating the SARS-CoV-2 S protein 67. After attachment and cleavage of the S protein, SARS-CoV-2 is internalized through endocytosis, and the viral genome is released from the endosome 8, 68, 69. Other host proteases, such as FURIN, are also thought to facilitate processing of the S protein 70. Therefore, the expression of host cell proteins that contribute to SARS-CoV-2 infection provides early clues to speculate on the liver tropism of SARS-CoV-2.

To assess the expression of SARS-CoV-2 entry factors in liver tissue and their distribution across cell types, single-cell RNA sequencing (scRNA-seq) analysis was performed based on published single-cell datasets 71-74. These studies indicate that ACE2 expression can be found on hepatocytes, but it is mainly expressed by liver cholangiocytes. This finding is consistent with the expression pattern of ACE2 in the liver and gallbladder in the Human Protein Atlas (https://www.proteinatlas.org/). In addition, TMPRSS2 and FURIN can also be detected in the liver and are broadly expressed rather than on specific cell types 72. Notably, entry of SARS-CoV-2 into cells may be dependent on the expression of TMPRSS2. As long as TMPRSS2 is present, low or barely detectable levels of ACE2 still support SARS-CoV-2 entry into cells 75. Therefore, one study analyzed the coexpression of ACE2 and TMPRSS2 based on three human liver single-cell transcriptomic datasets and found that very few hepatocytes (0.03-0.04%) coexpressed ACE2 and TMPRSS2 76. Moreover, another study showed that coexpression of ACE2 and TMPRSS2 only occurred in TROP2^+^ cholangiocyte-biased progenitor cells 77. These scRNA-seq-based studies suggest that SARS-CoV-2 might preferentially infect cholangiocytes and cause damage to the bile ducts, but it is difficult to explain why the pattern of liver injury in COVID-19 patients is mainly hepatocellular rather than cholestatic 57. Notably, transmission electron microscopy (TEM) revealed the presence of intact SARS-CoV-2 virus particles in the cytoplasm of hepatocytes from patients with COVID-19, which is direct evidence of SARS-CoV-2 infection of hepatocytes 55. Therefore, analysis of the expression of these host genes may underestimate the hepatic tropism of SARS-CoV-2 due to technical limitations of scRNA-seq.

Experimental cell and organoid models are of great significance for exploring the tropism of SARS-CoV-2 to different types of cells in the liver and its mechanism. It has been reported that the complete SARS-CoV-2 life cycle is observed in both the liver cancer cell lines Huh-7 and HepG2, supporting the infection and replication of the virus in hepatocytes 78-80. In addition, human primary hepatocytes and pluripotent stem cell-derived liver organoids also exhibit a permissive effect of hepatocytes for SARS-CoV-2 81. These studies provide favorable evidence for the hepatic tropism of SARS-CoV-2, suggesting that SARS-CoV-2 infection may directly lead to hepatocyte damage. Consistent with scRNA-seq data from liver tissue, human liver ductal organoids expressed ACE2 and TMPRSS2 and were infected by SARS-CoV-2, which impaired the function of cholangiocytes 82. As current evidence has not found other resident cells in the liver, such as hepatic stellate cells and Kupffer cells, that express ACE2 56, 76, 83, it is suggested that SARS-CoV-2 mainly enters and infects hepatocytes and cholangiocytes, causing liver dysfunction.

## Interplay between MAFLD and COVID-19

**Figure 1:**
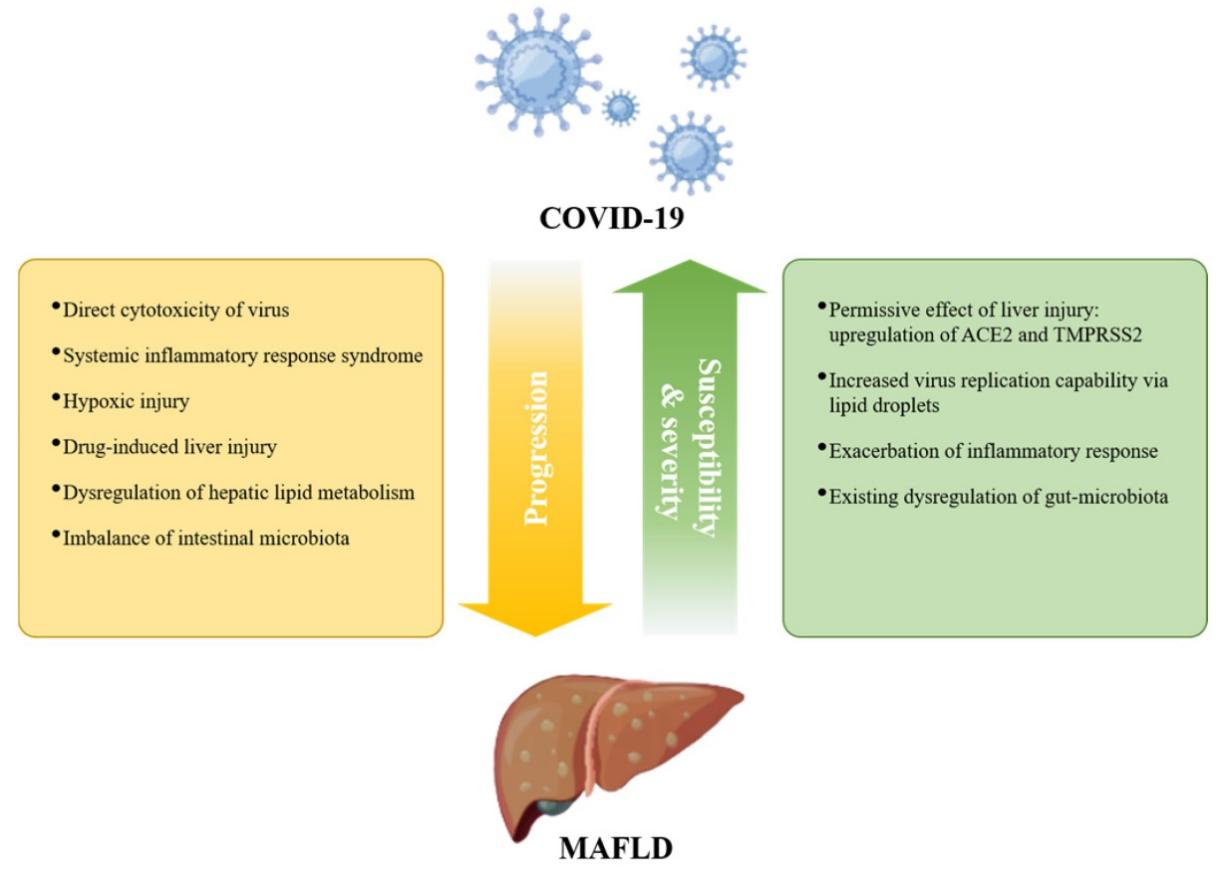
** Interplay between COVID-19 and MAFLD.** MAFLD may increase the susceptibility and severity of severe COVID-19; in turn, COVID-19 may promote the progression of preexisting MAFLD.

It has been shown that the presence of preexisting liver disease affects the prognosis of COVID-19 59, but the impact of MAFLD on COVID-19 progression has been controversial 84-86. Since MAFLD is a chronic disease affected by multiple factors and other metabolic dysfunctions, this inconsistency may be due to the extensive interaction of MAFLD with other metabolic comorbidities. Such confounding factors, as well as differences in diagnostic criteria, may lead to difficulties in analyzing risk factors for the COVID-19 pandemic 47, 87. Nonetheless, obesity, hyperglycemia/diabetes, and cardiovascular disease have been identified as distinct risk factors for adverse clinical outcomes in COVID-19 44, 62, 88. The incidence of MAFLD in patients with COVID-19 is higher than that in the general population 89, 90, suggesting that MAFLD contributes to enhanced susceptibility to COVID-19. Furthermore, the organoid culture system provides direct evidence for NASH-enhanced permissive effects for SARS-CoV-2 91. ACE2 was upregulated upon experimental liver injury 92, 93, and hepatic ACE2 and TMPRSS2 expression were increased in MAFLD patients 94. These findings possibly provide a mechanistic explanation for the increased susceptibility of MAFLD patients to SARS-CoV-2 (Figure 1).

Given the central role of the liver in the production of albumin, acute-phase reactants, and coagulation factors, liver dysfunction may affect multisystem manifestations of COVID-19, such as acute respiratory distress syndrome (ARDS), coagulation disorders, and multiorgan failure 6, 8, 24, 95-97, thereby exacerbating symptoms of COVID-19. MAFLD is an independent risk factor for severe COVID-19 in patients aged less than 60 years 57, 98-100. MAFLD patients with coexisting obesity had a more than 6-fold increased risk of severe COVID-19 101, and further studies indicated that the association of MAFLD with COVID-19 severity is independent of obesity or diabetes 102, 103. In addition, one recent retrospective study with 359 COVID-19 patients found that the death rate and intubation rate were significantly higher in MAFLD patients than in the control group 104. Liver function abnormalities and a longer viral shedding time caused by MAFLD contribute to COVID-19 progression 57, and the effect is more pronounced in MAFLD patients with advanced fibrosis 105-109. Furthermore, an increased neutrophil-to-lymphocyte ratio (NLR) in MAFLD may exacerbate SARS-CoV-2-induced inflammatory storms, thereby aggravating COVID-19 symptoms 110.

However, a recent study demonstrated that while MAFLD may be associated with severe COVID-19 at the population level, MAFLD is not a causal risk factor for severe COVID-19 based on two-sample Mendelian randomization (TSMR) analysis 111. The authors mentioned that their results may be limited by the small sample size and other unknown clinical covariates 111. In addition, TSMR studies consider the lifetime effects of genetic variation rather than short-term measurements of specific parameters, and in some cases Mendelian randomization and its biological plausibility may not hold. These may be the reasons for the inconsistent results. Nevertheless, we need to draw more attention to the association of MAFLD and its comorbidities with COVID-19 progression (Figure 1).

In turn, SARS-CoV-2 infection also aggravates MAFLD progression (Figure 1). Studies have shown that SARS-CoV-2 promotes metabolic complications at the systemic and organ levels, including hyperglycemia, hypertension, and low high-density lipoprotein cholesterol (HDL-C), in patients without preexisting metabolic disease 112, 113. A study describing the clinical characteristics of patients with combined MAFLD and COVID-19 found more severe liver injury in MAFLD patients with COVID-19 than in uninfected MAFLD patients 114. Moreover, another follow-up of 235 discharged patients with COVID-19 found that the prevalence of MAFLD at follow-up was 55.3%, which was greater than the prevalence of MAFLD at admission (37.3%) 115. Therefore, special attention should be given to the management of patients with MAFLD during the COVID-19 pandemic. Next, we will focus on summarizing the possible molecular mechanisms by which COVID-19 affects MAFLD.

## Potential mechanism for COVID-19 promoting MAFLD progression

### Direct cytotoxicity of virus

SARS-CoV-2 in the intestinal lumen can be transferred to the liver through portal blood flow, enter cells through the ACE2 receptor and actively replicate to induce direct damage to the liver 33. Studies have shown that SARS-CoV-2 can induce apoptosis of infected cells 116, 117. In situ hybridization analysis has revealed SARS-CoV-2 virions in the vascular lumen and portal endothelial cells of COVID-19 liver specimens 58, and TEM also found the presence of intact virions in the cytoplasm of hepatocytes 55. Liver injury in COVID-19 patients is associated with the SARS-CoV-2 viral load, suggesting that the persistence of the virus may also cause direct damage to the liver 118. It is known that impaired mitochondrial activity is associated with the pathogenesis of NAFLD/NASH 119. Based on transcriptomic analysis and ultrastructural examination, SARS-CoV-2 infection suppressed hepatic mitochondrial activity and caused obvious mitochondrial swelling of hepatocytes 55, 120, strongly suggesting that SARS-CoV-2 directly causes cytopathic effects and contributes to MAFLD progression.

### Systemic inflammatory response syndrome (SIRS)

There is increasing evidence that patients with severe COVID-19 have cytokine storm syndrome 121. Elevated inflammatory biomarkers, such as C-reactive protein (CRP), ferritin, lactate dehydrogenase (LDH), D-dimer, IFN-γ, TNF-α, interleukin (IL)-2, IL-6, and monocyte chemoattractant protein-1 (MCP1), have been reported in severe COVID-19 patients 25, 122, 123. MAFLD is thought to be related to innate immune-mediated inflammation. According to recent knowledge about cytokine storm syndrome in COVID-19 patients, we propose that MAFLD may be exacerbated by the inflammatory response of COVID-19. First, IL-6 levels are elevated in MAFLD patients and associated with hepatic steatosis 124, 125. After SARS-CoV-2 infection, granulocyte-macrophage colony-stimulating factor (GM-CSF), produced by pathogenic T-cell activation, can activate CD14^+^CD16^+^ inflammatory monocytes to produce large amounts of IL-6 and other proinflammatory factors, which may contribute to MAFLD progression 126. Second, MCP1 is also involved in aggravating steatohepatitis 127. A recent study identified specific CD16^+^ T cells with enhanced cytotoxicity in severe COVID-19, which promote microvascular endothelial cell injury and the release of chemokines, including MCP1 128. Third, the liver contains the largest population of all tissue-resident macrophages (Kupffer cells), which contribute to the development of liver disease. In response to SARS-CoV-2 infection, lung resident macrophages can activate inflammasomes, secrete IL-1 and IL-18, and undergo pyroptosis, thereby leading to a hyperinflammatory state 129, indicating that the inflammasome-activated inflammatory response in Kupffer cells may also promote the progression of MAFLD. Fourth, cGAS-STING signaling-induced IFN has been shown to play an essential role in the development and progression of MAFLD 130. SARS-CoV-2 infection induces cGAS-STING activation in endothelial cells through mitochondrial DNA release, resulting in cell death and type I IFN production 131. As noted above, SARS-CoV-2 infection caused mitochondrial swelling in hepatocytes, suggesting that activation of cGAS-STING signaling may exacerbate MAFLD in COVID-19 patients.

Notably, autoimmune hepatitis symptoms have been described following SARS-CoV-2 vaccination 132-134. SARS-CoV-2 infection elicits not only innate but also adaptive immune responses. It has been reported that commercial monoclonal antibodies against SARS-CoV-2 spike protein or nucleoprotein can cross-react with human tissue antigens 135, 136, which suggests that immune cross-reactivity may cause SARS-CoV-2 infection or vaccine-induced autoimmunity. A recent study also found that SARS-CoV-2-specific activated CD8 T cells were enriched in the liver after SARS-CoV-2 vaccination, thereby leading to hepatitis 137. These studies suggest that in addition to the innate immune response caused by SARS-CoV-2 infection, the adaptive immune response may also contribute to the severity of MAFLD.

### Hypoxic injury

In addition to SIRS, severe COVID-19 patients often experience other severe complications, including ARDS and multiple organ failure, which can cause hypoxia and shock, leading to liver ischemia and hypoxia 138. Hepatic hypoxia in COVID-19 patients may lead to increased levels of ROS, NO derivatives, and hypoxia-inducible factors (HIFs) 33, 139, 140. Increased HIFs in COVID-19 patients can promote obesity and insulin resistance 141, which are important risk factors for MAFLD. HIF-1α has been reported to have the potential to promote MAFLD development 142. Moreover, hypoxia-induced HIF-2α overexpression aggravated MAFLD progression by inhibiting fatty acid β-oxidation and inducing lipogenesis in the liver through PPARα 143. Therefore, such changes in severe COVID-19 patients may further exacerbate the progression of MAFLD.

### Drug-induced liver injury (DILI)

At the beginning of the COVID-19 outbreak, there was no evidence-based drug treatment. Various drugs are used clinically to combat COVID-19, such as antiviral drugs (remdesivir, lopinavir/ritonavir), antibiotics (macrolides), antimalarial/antirheumatic drugs (hydroxychloroquine), immunomodulatory drugs (corticosteroids, tocilizumab) and anti-fever medications (acetaminophen), but many of these drugs have been considered to be hepatotoxic 144, 145. The use of lopinavir and ritonavir has been reported to be independently associated with elevated ALT/AST in patients with COVID-19 146. The presence of underlying metabolic abnormalities and MAFLD can contribute to DILI 30, 57; on the other hand, MAFLD also aggravates the hepatotoxicity of drugs such as acetaminophen, promoting the progression of MAFLD to NASH and even cirrhosis 144. Corticosteroid, the recommended drug for severe COVID-19, is also clearly associated with steatosis or glycogen deposition 145. Therefore, for patients with chronic liver disease, the risk of liver injury should be considered when choosing medications to treat COVID. The use of drugs with high hepatotoxicity may promote the progression of MAFLD.

### Dysregulation of hepatic lipid metabolism

In the infectious state, activated innate immunity not only directly triggers and amplifies liver inflammation but also interferes with the regulation of lipid metabolism, thereby promoting the development of liver fibrosis in MAFLD/NASH patients 147. Proteomic and metabolomic analyses revealed dyslipidemia in COVID-19 patients, such as lipid accumulation and downregulation of apolipoproteins 148, 149. In turn, it was found that SARS-CoV-2 infection can modulate pathways of lipid synthesis and uptake, thereby increasing lipid droplet (LD) accumulation in human cells. Meanwhile, SARS-CoV-2 can highjack LDs to enhance its replication capacity 150. Mechanistically, recent studies have indicated that ACE2 plays an important role in maintaining metabolic homeostasis. SARS-CoV-2 infection impairs ACE2 expression, which in turn induces metabolic abnormalities 151. This metabolic imbalance caused by ACE2 impairment may promote MAFLD progression in COVID-19 patients.

### Imbalance of intestinal microbiota

The gastrointestinal tract is not only the primary habitat for human microbiota but is also a target for SARS-CoV-2 infection, as it expresses high levels of ACE2 and TMPRSS2 152. There is increasing evidence that disruption of the microbiota balance during COVID-19 is associated with disease severity and mortality 153. The intestine has an interaction with the liver through the liver-gut axis, and the intestinal microbiota plays an important role in MAFLD progression 19, 154, suggesting that intestinal dysbiosis may contribute to the severity of MAFLD during COVID-19.

Based on a pilot study of 15 patients with COVID-19, alterations in intestinal microbiota and their association with susceptibility to severe disease have been reported 155. Compared to the microbiota of the healthy group, anti-inflammatory bacteria such as *Eubacterium ventriosum*, *Faecalibacterium prausnitzii*, *Roseburia*, and *Lachnospiraceae* decreased, but opportunistic pathogens* Clostridium hathewayi*, *Actinomyces viscosus*, and *Bacteroides nordii* increased in patients with COVID-19. A study of 30 COVID-19 patients revealed that COVID-19 patients have a higher abundance of opportunistic pathogens, such as *Streptococcus*, *Rothia*, *Veillonella* and *Actinomyces*, and a lower abundance of beneficial symbionts 156. Another cohort (62 COVID-19 patients) also found that *Roseburia* and *Faecalibacterium* decreased, while* Clostridium* and* Streptococcus* increased in COVID-19 patients 157. Furthermore, a larger cohort (100 COVID-19 patients) from two hospitals showed that several gut commensals with known immunomodulatory potential, such as *Faecalibacterium prausnitzii*, *Eubacterium rectale* and several bifidobacterial species, were depleted in patients, and this perturbed composition in patients with COVID-19 is concordant with disease severity 158. These studies indicate enrichment of opportunistic pathogens and depletion of commensals in the intestinal microbiota of COVID-19 patients.

**Figure 2:**
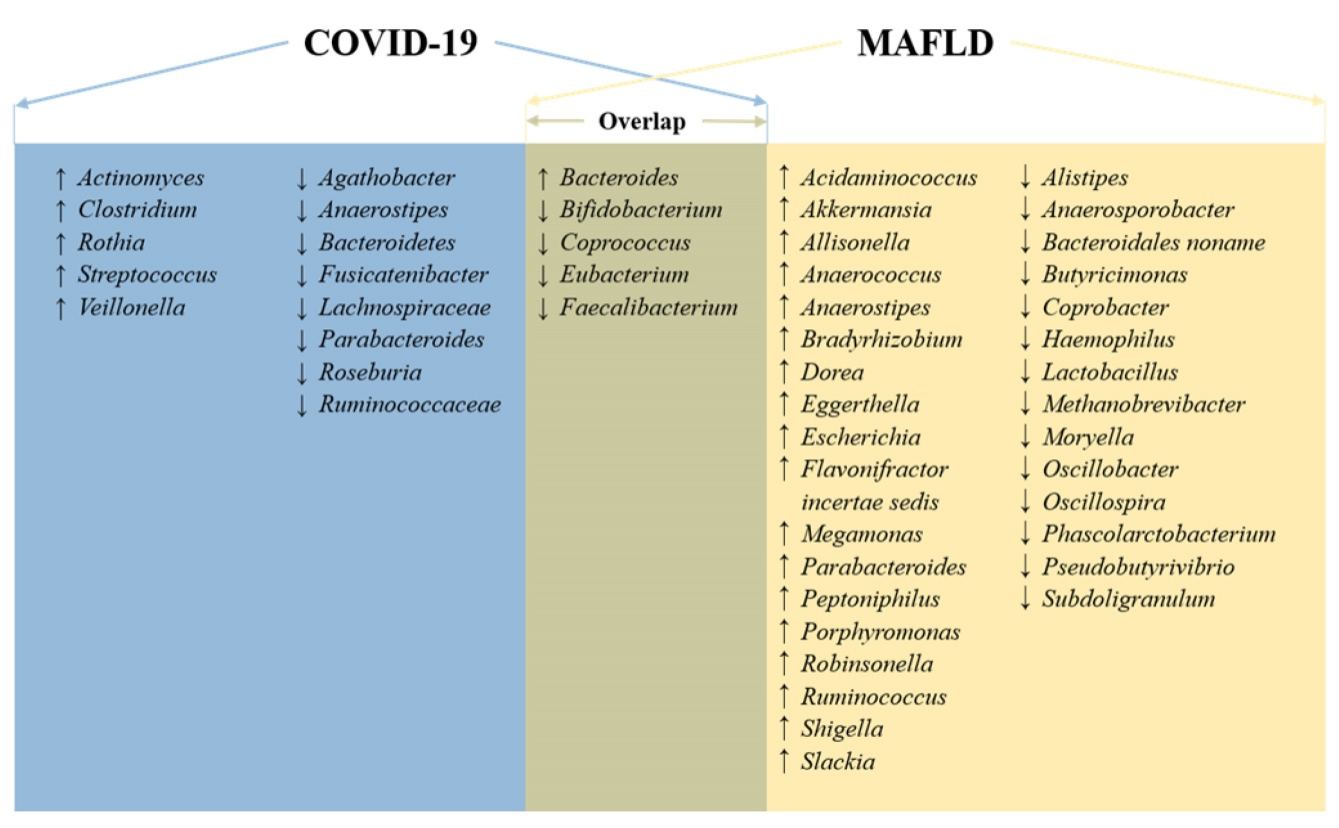
** Overlapping microbiota and general signatures in COVID-19 and MAFLD.** Microbiota with an up arrow was found to be more abundant in patients with MAFLD or COVID-19 than in healthy individuals, and vice versa.

According to a recent review about the relationship between intestinal microbiota and NAFLD in humans, we found that both diseases shared common altered bacteria, such as *Bacteroides*, *Eubacterium*, *Faecalibacterium*, *Coprococcus*, and *Bifidobacterium* (Figure 2) 159. Therefore, intestinal dysbiosis can cause intestinal and liver inflammation through translocation of endotoxins and bacteria due to increased intestinal permeability. Then, intestinal dysbiosis-induced hepatic inflammation, together with the SIRS mentioned above, further exacerbates MAFLD. 

Moreover, intestinal commensal-derived metabolites are involved in the development and progression of MAFLD, fibrosis and cirrhosis 159. For instance, lactate, ethanol, lipopolysaccharide (LPS), and trimethyl N-oxide (TMAO) can accelerate MAFLD progression, while short-chain fatty acids (SCFAs), such as acetate, proprionate, and butyrate, can have anti-inflammatory properties, thus preventing the progression of MAFLD 159. According to a hamster model, the abundance of bacteria known to produce SCFAs, such as *Ruminococcaceae* and *Lachnospiraceae,* was reduced following SARS-CoV-2 infection, as was the amount of systemic SCFAs 160. Although this was obtained from animal experiments, it reflects that alterations in microbial metabolites caused by disturbances in the intestinal microbiota can affect the risk of COVID-19. On the other hand, intestinal dysbiosis is also present in MAFLD, which leads to changes in the levels of bacterial metabolites such as butyrate and bile acids through the liver-gut axis 161. Consequently, there is an increased risk of local and systemic low-grade inflammation and decreased anti-inflammatory capacity in the intestine, thereby increasing the severity of COVID-19.

**Figure 3:**
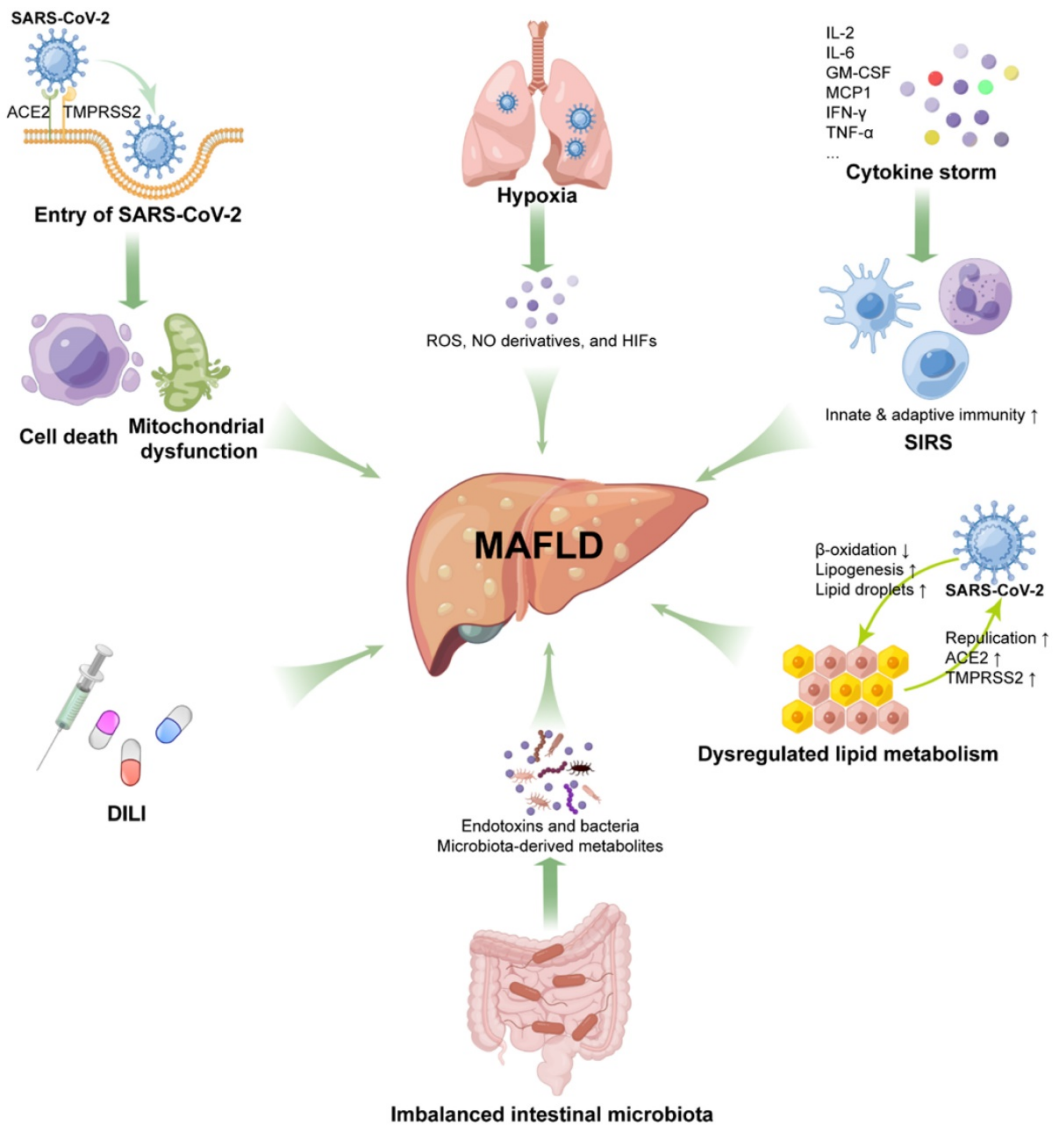
** Potential mechanisms for MAFLD progression under SARS-CoV-2 infection.** Direct cytotoxicity of SARS-CoV-2, hypoxia-mediated liver injury, drug-induced liver injury, systemic inflammatory response syndrome (SIRS), dysregulated lipid metabolism, and imbalanced intestinal microbiota are involved in MAFLD initiation and progression.

In short, SARS-CoV-2 infection can promote the occurrence and development of MAFLD through multiple pathways (Figure 3). As the research moves along, more underlying mechanisms will be discovered.

## Conclusions and perspectives

Numerous clinical studies have indicated that liver dysfunction is a common feature in COVID-19 patients and correlates with disease severity, suggesting an interaction between MAFLD and COVID-19, not only in that MAFLD contributes to the susceptibility and severity of COVID-19 but also in that COVID-19 may induce and accelerate the progression of MAFLD (Figure 1). Most existing studies are retrospective studies, which may be affected by confounding factors such as small sample size, limited survey area, and differences in diagnostic criteria. A multisample, multicenter long-term follow-up study is also required to explore the long-term consequences of the potential association between MAFLD and SARS-CoV-2 infection. Undeniably, multiple basic and clinical studies have demonstrated the tropism of SARS-CoV-2 to the liver. While human genome integration of SARS-CoV-2 is still under debate 162, 163, we cannot ignore the long-term effects of its infection on our bodies, especially the liver. This review summarizes multiple potential mechanisms by which COVID-19 drives the progression of MAFLD, namely, direct damage from viral infection, systemic inflammatory response, hypoxic injury, drug-induced liver injury, dysregulation of liver metabolism, and intestinal dysbiosis. As the epidemic continues, we should strengthen the monitoring and management of COVID-19 patients with MAFLD.

The generally recommended management for patients with MAFLD is similar to that of healthy individuals. Meanwhile, enhanced personal protection 164 along with good lifestyle habits 165 (including weight loss advice, nutritional guidance, and diabetes management) may reduce the chance and severity of COVID-19 infection and slow the progression of liver injury. Considering the possible increased risk of severe COVID-19, the European Association for the Study of the Liver (EASL) recommends that all MAFLD patients infected with SARS-CoV-2 should receive standardized and timely diagnosis and treatment as soon as possible 166. In addition, MAFLD patients often have other metabolic comorbidities, such as hyperglycemia, obesity, and hypertension, which are risk factors affecting the prognosis of COVID-19. Monitoring and management of these metabolic disorders can minimize the risk of a poor prognosis in MAFLD patients with SARS-CoV-2 infection 167. As new therapeutic drugs are being developed for COVID-19, potential hepatotoxic drug-drug interactions need to be assessed, and timely adjustment of medications for patients with MAFLD may also reduce the risk of adverse outcomes.

In conclusion, the COVID-19 pandemic continues, and new variants of SARS-CoV-2, such as Omicron BA.1 and BA.2, are spreading more rapidly and aggressively. Since MAFLD is considered a growing chronic pandemic affecting a quarter of the global adult population, we need to pay more attention to COVID-19 patients with MAFLD and develop new therapeutic strategies, such as imatinib and methazolamide treatment 151, to improve metabolic complications caused by COVID-19. Moreover, we need to establish a long-term follow-up program to monitor the prognosis and incidence of liver cancer in these patients.

## Figures and Tables

**Table 1:** Liver biochemistry abnormalities of COVID-19 patients

Study	Region	Sample collection date	Sample size (n)	Elevated ALT	Elevated AST	Elevated ALP	Elevated GGT	Elevated TBIL
Guan^48^	Nationwide, China	2019/12/11-2020/01/29	722-757	158/741 (21.3%)	168/757 (22.2%)	NA	NA	76/722 (10.5%)
Zhang^34^	Wuhan, China	2019/12/29-2020/02/16	267	49 (18.4%)	76 (28.5%)	NA	NA	NA
Cai^35^	Shenzhen, China	2020/01/11-2020/02/21	417	54 (12.9%)	76 (18.2%)	101 (24.2%)	68 (16.3%)	99 (23.7%)
Xu^36^	Shanghai, China	2020/01/20-2020/10/20	1003	295 (29.4%)	176 (17.5%)	26 (2.6%)	134 (13.4%)	40 (4.0%)
Ding^37^	Wuhan, China	2020/01/28-2020/04/25	2073	501 (24.2%)	545 (26.3%)	165 (8.0%)	443 (21.4%)	71 (3.4%)
Fu^38^	Wuhan, China	2020/02/01-2020/02/20	482	96 (19.9%)	98 (20.3%)	NA	NA	23 (4.8%)
Lv^39^	Wuhan, China	2020/02/05-2020/03/23	2912	662 (22.7%)	221 (7.5%)	135 (4.6%)	536 (18.4%)	52 (1.8%)
Benedé-Ubieto^40^	Madrid, Spain	2020/02/25-2020/04/23	799	204 (25.73%)	446 (49.17%)	186 (24.21%)	270 (34.62%)	NA
Richardson^43^	New York, America	2020/03/01-2020/04/04	5700	2176 (39.0%)	3263 (58.4%)	NA	NA	NA
Weber^41^	Munich, Germany	2020/03-2020/07	217	59 (27.2%)	91 (41.9%)	22 (10.1%)	80 (36.9%)	10 (4.6%)
